# Herpes simplex encephalitis in adult patients with MASP-2 deficiency

**DOI:** 10.1371/journal.ppat.1008168

**Published:** 2019-12-23

**Authors:** Stéphanie Bibert, Jocelyne Piret, Mathieu Quinodoz, Emilie Collinet, Vincent Zoete, Olivier Michielin, Rafik Menasria, Pascal Meylan, Titus Bihl, Véronique Erard, Florence Fellmann, Carlo Rivolta, Guy Boivin, Pierre-Yves Bochud

**Affiliations:** 1 Infectious Diseases Service, Department of Medicine, University Hospital and University of Lausanne, Lausanne, Switzerland; 2 Research center in Infectious Diseases, CHU of Quebec and Laval University, Quebec city, Canada; 3 Department of Computational Biology, Unit of Medical Genetics, University of Lausanne, Lausanne Switzerland; 4 Ludwig Institute for Cancer research, University of Lausanne, Lausanne, Switzerland; 5 Molecular Modeling Group, Swiss Institute of Bioinformatics, Quartier Sorge, Génopode, Lausanne, Switzerland; 6 Department of Oncology, University Hospital and University of Lausanne, Lausanne, Switzerland; 7 Institute of Microbiology, Department of Laboratory Medicine, University Hospital and University of Lausanne, Lausanne, Switzerland; 8 Canton Hospital of Fribourg, Fribourg, Switzerland; 9 Department of Genetics, Laboratoire National de Santé, Dudelange, Luxembourg; 10 Department of Genetics and Genome Biology, University of Leicester, Leicester, United Kingdom; Louisiana State University Health Sciences Center, UNITED STATES

## Abstract

We report here two cases of Herpes simplex virus encephalitis (HSE) in adult patients with very rare, previously uncharacterized, non synonymous heterozygous G634R and R203W substitution in mannan-binding lectin serine protease 2 (*MASP2*), a gene encoding a key protease of the lectin pathway of the complement system. None of the 2 patients had variants in genes involved in the TLR3-interferon signaling pathway. Both *MASP2* variants induced functional defects *in vitro*, including a reduced (R203W) or abolished (G634R) protein secretion, a lost capability to cleave MASP-2 precursor into its active form (G634R) and an in vivo reduced antiviral activity (G634R). In a murine model of HSE, animals deficient in mannose binding lectins (MBL, the main pattern recognition molecule associated with MASP-2) had a decreased survival rate and an increased brain burden of HSV-1 compared to WT C57BL/6J mice. Altogether, these data suggest that MASP-2 deficiency can increase susceptibility to adult HSE.

## Author summary

Human herpes virus type 1 (HSV-1) infects a large number of individuals during their life, with manifestations usually limited to mild and self-limiting inflammation of the oral mucosa (cold sore). However, HSV-1 can cause a very severe disease of the brain called Herpes simplex encephalitis (HSE) in 1 out of 250’000–500’000 individuals per year. The reasons why HSV-1 can cause such a devastating disease in a very limited number of individuals are unknown. Increasing evidence suggests that susceptibility to HSE in children can results from genetic variations in the immune system, in particular in a viral detection pathway called the Toll-like receptor 3 (TLR3)–interferon (IFN) axis. Fewer data are available to explain HSE in adult patients. Here, we describe two adult patients with HSE who carry mutations in a gene called mannan-binding lectin serine protease 2 (*MASP2)*, which is part of an immune pathway different from the TLR3-IFN axis, called the lectin pathway of the complement system. We demonstrate that MASP2 mutations induce functional defects in immune defense against HSV-1 that prevent viral replication. Mice deficient in the lectin pathway have higher mortality compared to wild-type mice after HSV-1 infection. Altogether, our study suggests that susceptibility to HSE in adults relies of immune deficiencies that are different from those causing HSE in children.

## Introduction

Herpes simplex virus encephalitis (HSE) is the most common cause of sporadic fatal viral encephalitis in Western countries with a mortality rate of ~70% in the absence of antiviral treatment [1]. It occurs in 2–4 individuals per million and per year, with a bimodal age distribution including children, mainly as a result of primary infection [2, 3], and adults over the age of 50 years, as a result of reactivation from a latent infection [4].

Studies mainly performed in children revealed that the TLR3 pathway is essential and non-redundant for induction of IFN-α, β, λ and γ in the central nervous system (CNS) [5] and that the pathogenesis of HSE and its recurrence may result from single-gene inborn errors of TLR3/IFN pathway-mediated immunity [6–14]. Most of these result from autosomal-dominant variants with incomplete clinical penetrance [15], highlighting the importance of environmental, pathogen and additional host factors. Rare variants in the TLR3/IFN signaling pathway have also been identified in adults [16] confirming that impaired TLR3-mediated immunity may also increase susceptibility to HSE in this population. Nevertheless, two studies in adult patients identified variants in genes not directly involved in the TLR3/IFN pathway, indicating that other genes/pathways may also be associated with the development of HSE [17, 18].

We report here 2 cases of adult HSE in patients with very rare variants in mannan-binding lectin serine protease 2 (MASP-2), the gene encoding a protease considered as the central activator of the lectin pathway of the complement system (the most ancient, besides the classical and alternate pathways of complement activation), because of its ability to form complexes with several pattern recognition molecules including mannose-binding lectin (MBL), collectins (CL-L1 and CL-K1) and ficolins (FCN1, FCN2 and FCN3 also called M-, L- and H-ficolin respectively).

## Results

The presence of very rare and deleterious variants in innate immune genes was analysed in 15 HSE adult patients of European ancestry who did not present other unusual severe infectious diseases, compared to 294 controls of same ethnic background. Among the 950 innate immune genes tested, 33 presented at least one qualifying variant in one case of HSE and none was significant, following correction for multiple testing. However, we could identify 4 genes with suggestive evidence for enrichment, in qualifying variants in HSE cases vs. controls. None of these genes were related to the TLR3/IFN signaling pathway. We focused our analysis on one of them, namely the mannan-binding lectin serine protease 2 (MASP2).

MASP-2 is involved in the complement system as the central protein of the lectin pathway, which is triggered by specific recognition of pathogens. Notably, mice deficient in MASP2 or MBLs (two genes involved in the lectin pathway) were recently shown to have increased mortality compared to WT mice in a model of West Nile virus infection [19]. Specifically, two out of the 15 HSE individuals carried 2 different very rare and predicted to be deleterious variants compared to 5 of such variants in 294 controls (OR 8.26, individual P = 0.041). We confirmed the enrichment by comparing the same cases with gnomAD-NFE (Non-Finnish Europeans) and found a similar OR of 12.6 (p = 0.013). In contrast, no very rare non-synonymous variants in the MBL gene were observed among HSE cases. Because frequent MBL variants are strongly associated with lectin pathway activity, we compared their frequencies among HSE cases and controls. The proportion of frequent functional MBL variants (namely rs5030737, rs1800450, rs1800451, rs11003125, rs7096206, rs7095891 which form haplotypes HYPA, LYQA, LYPA, LXPA, HYPD, LYPB, LYQC, LYPD associated with low MBL serum concentration) was similar in both groups (OR = 0.83, 95% confidence interval 0.33–1.97, P = 0.8).

**Fig 1:**
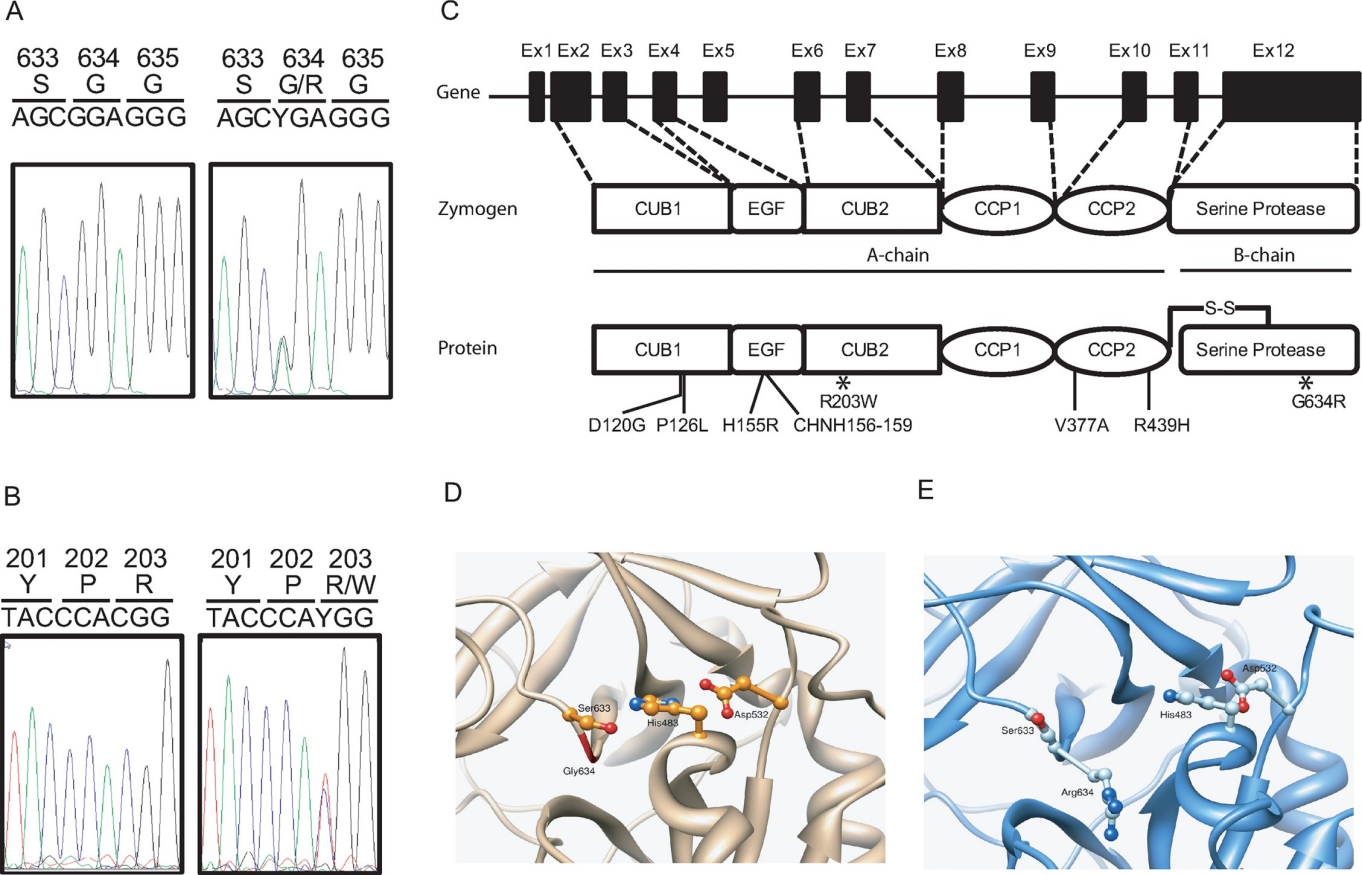
*MASP2* mutations in HSE patients. **A.** and **B.** Sanger sequencing of PCR products amplified from genomic DNA from control and patients carrying the G634R (A) or the R203W (B) mutation. **C.** Schematic representation of *MASP2* with exons (Ex) 1–12, zymogen and active protein featuring the different domains as well as the location of non-synonymous functional variants. MASP-2 is composed of well-defined domains comprising the CUB1 (C1r/C1s, Uegf and bone morphogenetic protein-1 domains), EGF (epidermal growth factor), CUB2, CCP1, CCP2 (complement control protein module 1 and 2) and serine protease. **D.** Experimental predicted 3D structure of MASP-2 in ribbon representation (PDB ID 1ZJK). The active catalytic triad Asp532, His483 and Ser633 is displayed in ball and stick. The backbone of residues 483, 532, 633 and 634 are colored in green. **E**. Last frame of one MD simulation of the G634R MASP-2 in the same orientation. Arg634 protrudes between His483 and Ser633, separating these two residues and impairing the catalytic triad.

Among the two HSE adult patients who have non-synonymous rare variants in *MASP2*, one was a female patient (P1) carrying a heterozygous NM_006610.3:c.1900G>A (genome build hg19) mutation resulting in a replacement of a glycine residue to an arginine residue at amino acid position 634 (NP006601.2) within the serine protease domain (G634R, **Fig 1A and 1C**). Patient P1 was hospitalized at the age of 60 for a generalized tonico-clonic seizures shortly preceded by important occipital headache (**Table 1**). She had no relevant co-morbidity except splenectomy during her childhood. Cerebrospinal fluid (CSF) real-time PCR was positive for HSV-1[20]. Magnetic resonance imaging showed lesions in the right temporal lobe. P1 was treated with acyclovir within hours after the onset of clinical symptoms and had full clinical recovery. P1 is the only member of this family to have developed HSE. Noteworthy, a 20 year old female patient in our exome sequencing dataset also carried the G634R mutation. She was hospitalized for a primary attack of multiple sclerosis. The medical chart review revealed that her CSF was tested positive for HSV-1. Although the clinical presentation was not typical for HSE, the patient received acyclovir treatment to cover a potential early form of HSE.

**Table 1:** Clinical features in 2 HSE patients.

	P1	P2
Age at presentation	60	24
Sex	Female	Male
Relevant comorbid conditions	Post-traumatic splenectomy	None
Clinical presentation		
Onset of symptoms	Acute	Progressive^1^
Fever	No	Yes
Headache	Yes	Yes
Photophobia	No	Yes
Seizures	Yes	Yes
Neuropsychological impairment	Confusion	Yes
Motor impairment	No	Yes
Family cases of HSE	None	None
HSV-1 serostatus before HSE	Unknown	Unknown
History of cold sores	None	None
Cerebrospinal fluid analysis		
PCR for HSV-1	Positive	Positive
Cells count (per mm3)	16	450
Neutrophils (%)	34	3
Lymphocytes (%)	51	72
Proteins (g/L)	0.67	0.96
Glucose (mmol/l)	5.3	3.6
Glucose (CSF/serum ratio)	0.31	0.54
Lactate (mmol/l)	2.9	NA
Cerebral imaging	Right temporal cortical lesions	Right fronto-parieto-temporal cortical lesions Major oedema
Acyclovir therapy	Yes	Yes
(Timing from clinical onset)	(Hours)	(Several days)
Complications/management	seizures	Intubation (15 days) Intracranial hypertension requiring craniotomy
Outcome	Full recovery	Major neurological sequelae

^1^ Headache, nightmares and delusional ideas reported over 5 days before hospitalization.

NA stands for not available

The second patient P2 had a NM_006610.3:c.607C>T heterozygous nucleotide replacement **(Fig 1B)** which results in the substitution of an arginine residue for a tryptophan residue at amino acid position 203 in the CUB2 domain (R203W, **Fig 1B and 1C**). P2 was a previously healthy 24 years old man who was hospitalized for fever with headaches, photophobia and vomiting. The patient already presented unusual headaches, nightmares and delusional ideas during the 5 days preceding hospitalization (**Table 1**). He developed a tonico-clonic seizures after lumbar puncture and subsequently underwent craniotomy due to cerebral herniation. He was treated with acyclovir. Real time CSF PCR was positive for HSV-1. Cranial imaging initially revealed a signal in the cortical region, with important right temporal oedema, which progressively evolved towards right cerebral ventricle dilatation. The patient had important neurological sequelae with severe neuropsychological impairment.

The G634R mutation (P1) is referenced in ExAC as rs532646305, with a MAF of 1.1x10^-4^ among non-finnish Europeans and is predicted to be deleterious by the Sorting Intolerant from Tolerant (SIFT) and Polymorphism Phenotyping 2 (PolyPhen-2) scores. Three and six Molecular Dynamic (MD) simulations were performed for the wild type and G634R MASP-2, respectively, each 140 ns in length. The MD simulations of the wild-type system showed that the catalytic triad D532, H483 and S633 appeared very stable, as could be expected. D532 accepted a strong and stable hydrogen bond from H483. The average minimal distance between the two residues in the last 50 ns of these MD simulations was 2.84 ± 0.02 Å. A weaker, yet stable, hydrogen bond also existed between S633 and H483, with an average minimal distance of 3.8 ± 0.3 Å over the last 50 ns of the 3 MD simulations. These three residues remained constantly in contact during the simulations **(S1 Fig, S2 Fig).** On the contrary, the MD simulations of the mutated MASP-2 consistently showed that the mutation of residue G634 to R disrupted the catalytic triad by projecting the new R634 side chain between S633 and H483 leading to a dramatic increase of the distance between S633 and H483 (6.0 ± 0.4 Å during the last 50 ns of the MD simulations) which may impair the activity of MASP-2 (**Fig 1D and 1E)**.

The R203W mutation (P2) is referenced in ExAC as rs373318594, with a MAF of 1.3x10^-4^ among NFE and is predicted to be deleterious by SIFT and PolyPhen-2 scores. No experimental structure is available for MASP-2 between residues 181 and 287, preventing any precise and relevant MD simulations of the wild type and mutated proteins. Sequence alignment of MASP-2 from 19 organisms, including *Homo sapiens*, shows that R203 belongs to a very conserved motif 199-PEYPxPYPK-207. Although R203 is not itself conserved, it is only replaced by polar residues in other species (**S3 Fig**). This is perfectly in line with the experimental 3D structures of MASP-1 in this region (47.7% sequence identity with MASP-2), which shows that the PEYPxPYPK-like motif (i.e. 200-PDFPxPYPK-208 in MASP-1) is essential for the structure of the CUB2 domain. R203 of MASP-2 is replaced by N204 in MASP-1. The latter belongs to a loop, faces the solvent, and possibly makes a hydrogen bond with D201 (corresponding to E200 in MASP-2), explaining that this position should be occupied by a polar residue. Replacing R203 in the related position of MASP-2 by a bulky, non-polar and aromatic tryptophan residue is expected to impact the stability of this region (**S4 Fig)**.

**Fig 2:**
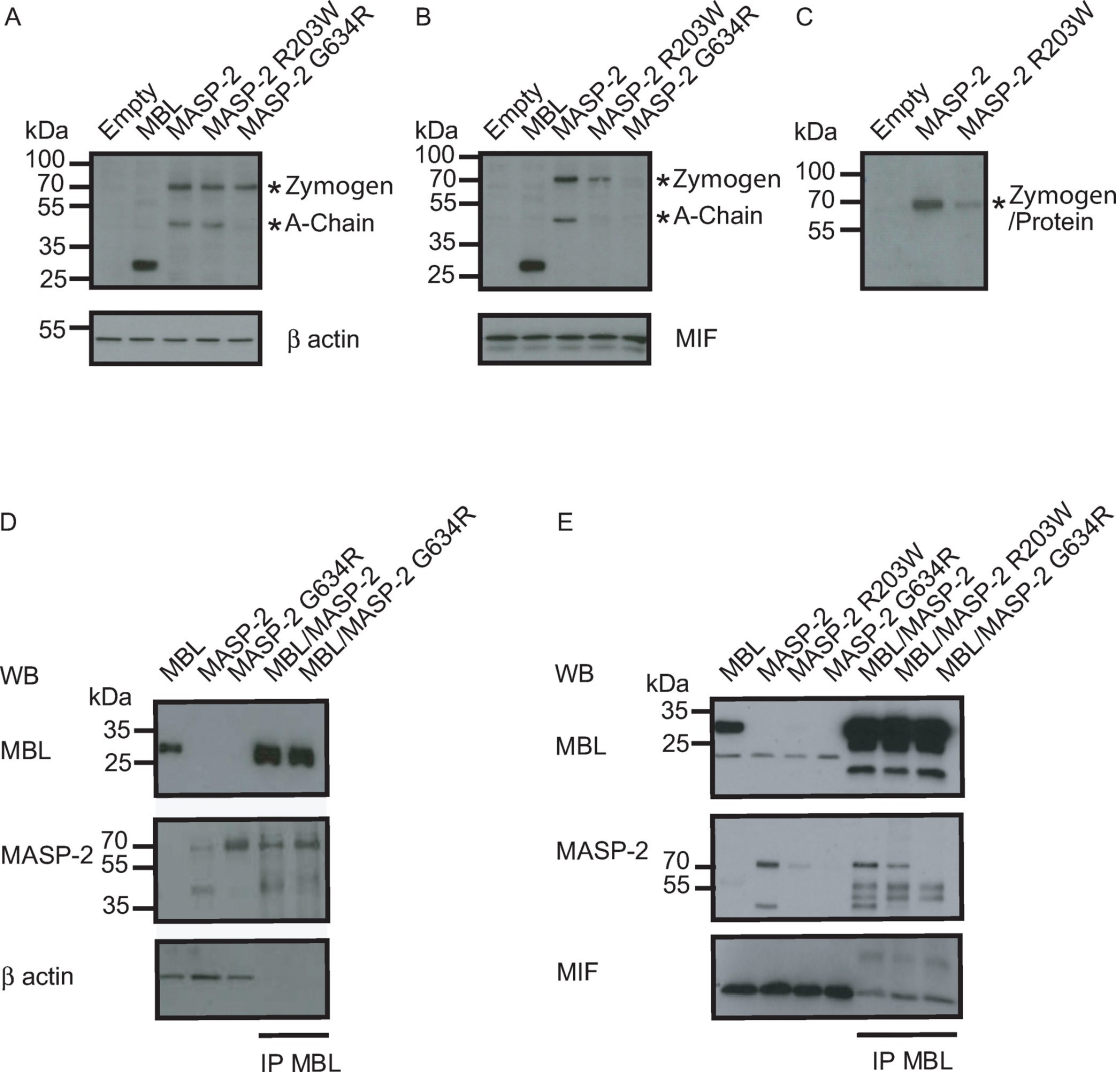
Biochemical characterization of G634R and R203W MASP-2 proteins. **A-C.** The ability of G634R and R203W MASP-2 proteins to be expressed, secreted and to auto-activate was determined by SDS-PAGE/immunoblotting under reducing (**A, B**) and non-reducing conditions (**C**) in cell lysates (**A**) and cell supernatant (**B, C**). Binding to recombinant MBL was determined in cell lysates (**D**) and cell supernatant (**E**) by co-immunoprecipitation experiments by using an antibody directed against MBL (IP). Housekeeping proteins β actin and macrophage migration inhibitory factor (MIF) were used as loading controls for cell lysates and cell supernatant respectively.

To biochemically characterize the heterozygous G634R and R203W variants, we cloned both wild-type and mutant cDNAs and expressed them in Hela cells, instead of directly using primary cells. The expression of MASP-2 in cell lysate **(Fig 2A)** and in the culture supernatant **(Fig 2B)** was analyzed by Western blotting. The precursor (zymogen) form of MASP-2 is represented by a 76kDa band. The active form is composed of two polypeptides linked by a disulfide bridge appearing as a single band (undistinguishable from zymogen) under non-reducing condition, and as two separate bands (a 52kDa A-chain and 24kDa B-chain) under reducing conditions. Since the epitope targeted by the Western blotting antibody was directed against the N-terminal part, both the zymogen and A chain, but not the B chain of MASP-2, could be detected. Under reducing conditions, the WT form of MASP-2 appeared as two bands in both the cell lysate and cell supernatant, suggesting that it is capable of auto-activation and can be secreted. In contrast, the G634R form appeared as a unique 76kDa band which is present in cell lysate, but not in cell supernatant, suggesting that it is not capable of self-activation and not secreted. As a control, under non-reducing conditions, the WT form appears as a unique 76kDa band (**Fig 2C**). Under reducing conditions, the R203W appears as two separate bands in cell lysate and cell supernatant, with lower intensity in the cell supernatant, suggesting that it is capable of self-activation, but is poorly secreted compared to the WT.

To compare the ability of the WT and mutant forms of MASP-2 to bind to MBL, the main lectin pathway pattern recognition molecules, lysates of cells expressing the WT and mutant forms were mixed with those expressing MBL. Complexes were then co-immunoprecipitated under non- reducing conditions with a MBL antibody. Both the WT and the G634R were capable to bind MBL expressed in cell lysates. Binding was observed for both the zymogen and the A-chain in the WT form of MASP-2, but only for the zymogen form of the G634R variant, further confirming its inability to auto-activate (**Fig 2D)**. In addition, MBL binding in supernatant was observed for the WT and the R203W variant of MASP-2, but not the G634R form, as a probable result of its reduced or abolished secretion (**Fig 2E).** Altogether, these observations suggest that the G634R and R203W forms of MASP-2 are both expressed and capable to bind MBL. Compared to WT form, the secretion of R203W is reduced, and that of G634R is almost abolished. In addition, the R203W form has a conserved ability to auto-activate, a capacity which is totally lost for G634R.

**Fig 3:**
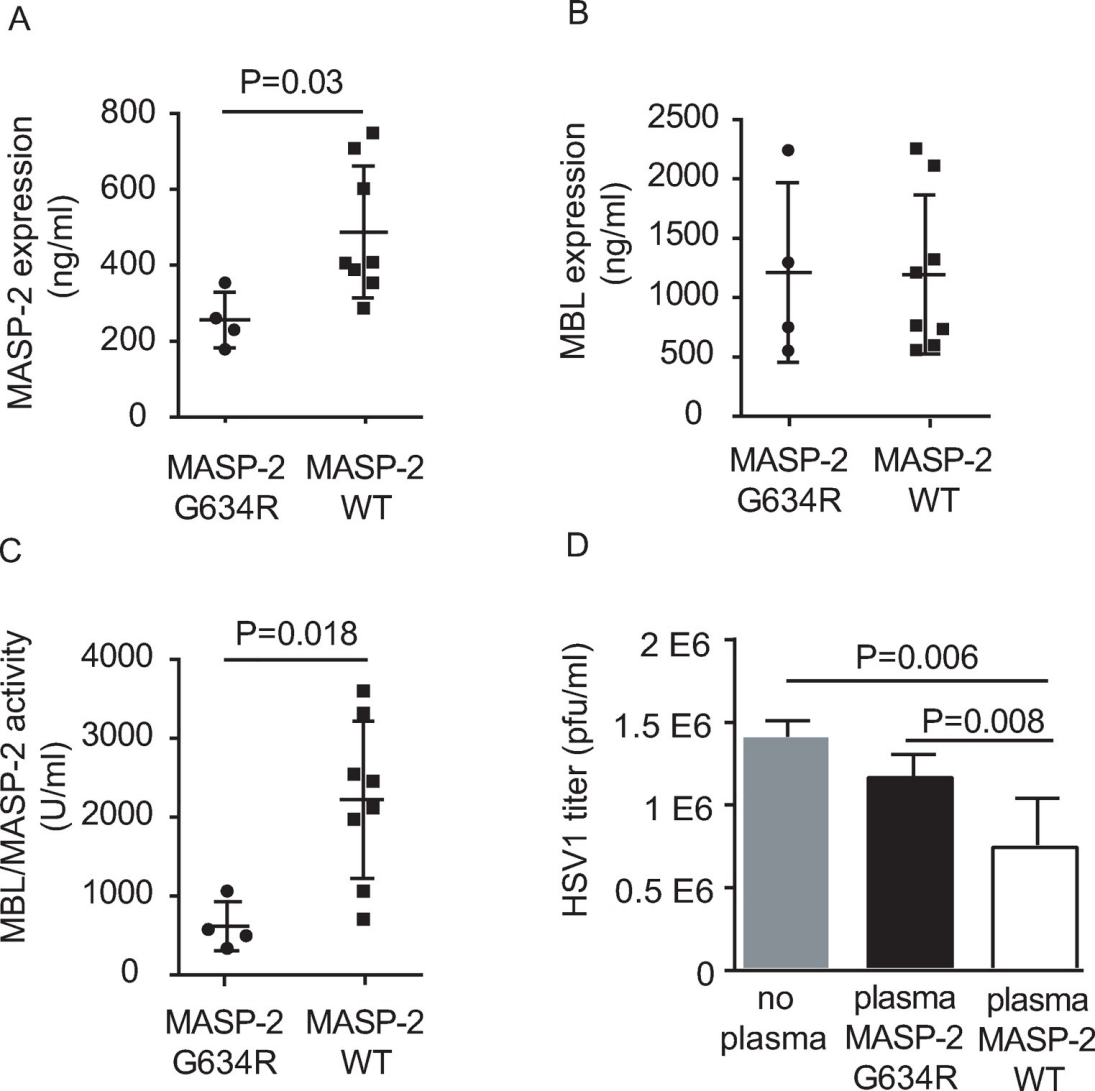
Functional characterization of G634R MASP-2 proteins. **A.** MASP-2 amount was evaluated in plasma of patient/family individuals carrying the G634R mutation (N = 4) as well as in WT individuals (N = 8). Each G634R individual was individually matched with 2 WT individuals having similar MBL levels (**B**) and the activity of MBL/MASP-2 complexes was determined by ELISA (**C**). **D.** Virus neutralization assay on plasma from both G634R and WT individuals. Virus titer determination was based on the presence of cytopathic effects on Vero cells. Statistical analyses were performed using an unpaired Student t-test.

To functionally characterize the consequence of heterozygous G634R carriage *in vivo*, the levels of MASP-2 (**Fig 3A)** and the MASP-2 activity of the MBL/MASP-2 pathway of the complement (**Fig 3C)** were analysed in plasma from G634R heterozygous carriers (N = 4) and WT controls (N = 8). MBL/MASP-2-induced complement activation was measured by the ability of plasma from G634R and WT individuals to deposit C4b on mannan-coated microtiter wells. In order to avoid a bias due to differential MBL expression, each G634R carrier was individually matched with 2 WT controls having comparable MBL levels (+/- 50ng/ml, **Fig 3B)**. As ficolins do not bind to mannan, all changes in the amount of C4 cleavage depends on MASP-2. We observed that the level of MASP-2 and C4b deposition activity of the MBL/MASP-2 complexes were both significantly lower in G634R individuals compared to WT individuals. In order to analyse the antiviral role of MASP-2, we measured titers of HSV-1 after incubation of the virus with plasma from G634R individuals and WT individuals **(Fig 3D)**. Plasma was previously depleted from antibodies in order to measure the direct effect of MASP-2 on HSV-1 neutralization [21] and to account for the absence of antibodies in non-inflamed meninges [22]. Plasma from heterozygous G634R carriers had a significant decreased ability to neutralize HSV-1 compared to plasma from WT controls. Thus, heterozygosity for the G634R *MASP2* allele confers autosomal dominant hyporesponsiveness to HSV-1 in plasma.

**Fig 4:**
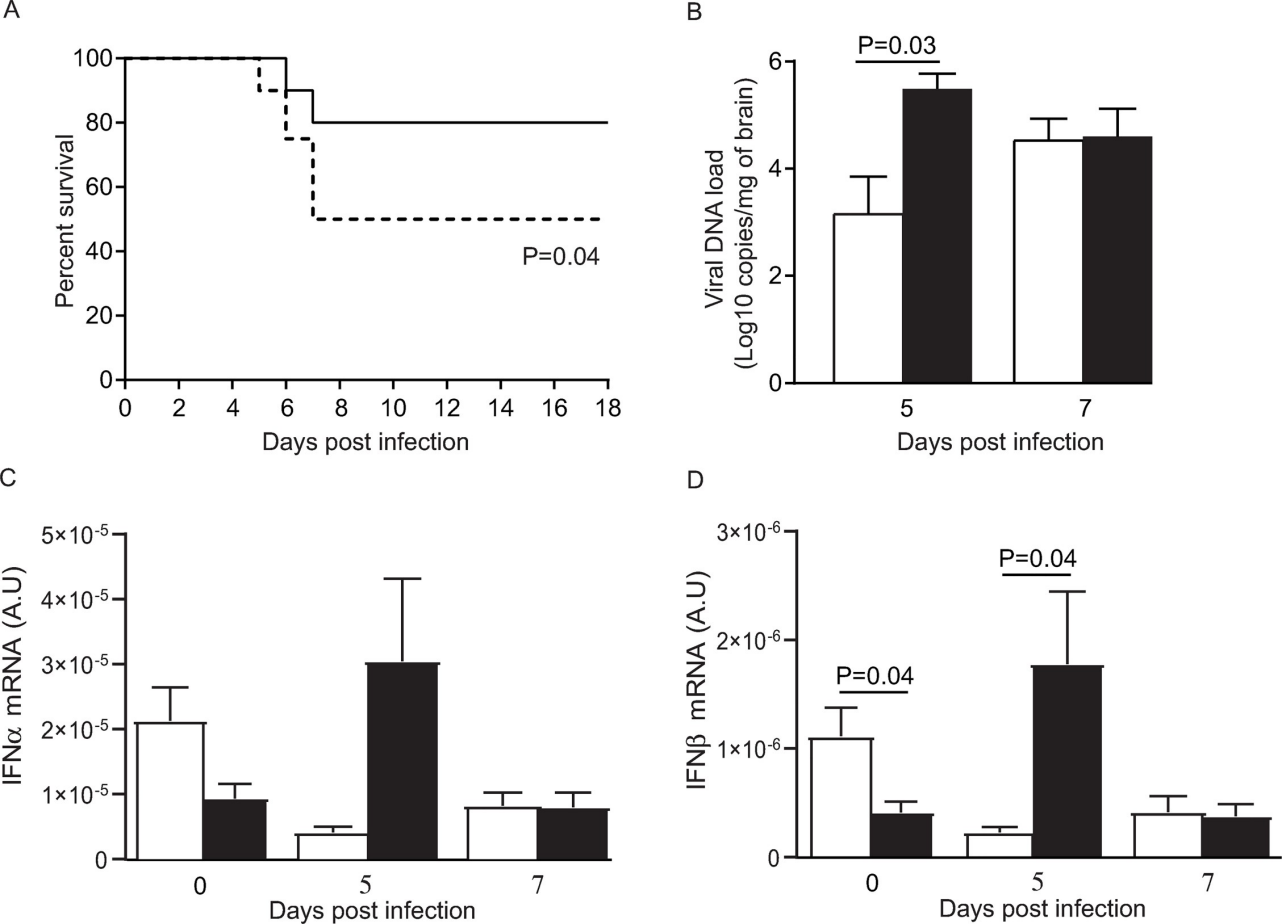
Impact of MBL deficiency in HSV-1 infected mice. **A.** Survival rates of WT and MBL-null (dotted line) mice. Results represent combined data from two separate experiments with 10 WT and 10 MBL-null mice each. Statistical analyses were performed using a log-rank (Mantel-Cox) test. **B**. Viral load in brain homogenates of WT (white) and MBL-null (black) mice. Results are mean ± standard error of the mean of 3–5 mice per group and per time-point. Statistical analyses were performed using an unpaired Student t-test. **C, D.** IFNα (C) and IFNβ (D) mRNA levels normalized to those of 18S mRNA, in brain homogenates of WT (white) and MBL-null (black) mice on days 0, 5 and 7 post-infection. Results represent the mean ± standard error of the mean of 3–5 mice per group and per time point. Statistical analyses were performed using an unpaired Student t-test. A.U. stands for arbitrary units.

MASP-2 is considered as the central activator of the lectin pathway of the complement system. To evaluate the role played by the lectin pathway in the pathogenesis of HSE, we used a murine model induced by intranasal HSV-1 injection. The survival of WT C57BL/6J mice was compared to that of mice deficient in MBL (MBL-null), as a surrogate marker for the MASP-2-dependent complement activation. MBL-null mice had significantly lower survival rates than WT mice (P = 0.04, **Fig 4A, S5 Fig)** suggesting that the lectin pathway is important for survival of infected mice. The role of the lectin pathway in controlling viral replication during HSE was further evaluated by measuring the viral DNA load in brain homogenates **(Fig 4B)**. The viral genome copies were significantly higher in brain homogenates of MBL-null than in WT animals on day 5 (P = 0.03), which corresponds to the peak of infection, but not on day 7 post-infection (P = 0.35). This suggests that the lectin pathway contributes to control HSV replication during HSE. This was confirmed by the expression level of IFN-α which tended to be higher on days 5 and 7 post-infection (P = 0.06) (**Fig 4C**) and that of IFN-β which was significantly increased compared to WT on day 5 (P = 0.04) but not on day 7 post-infection (P = 0.06, **Fig 4D**). Altogether, these data suggest that the MBL/lectin pathway contributes to HSV-1 immunity in brain.

**Fig 5:**
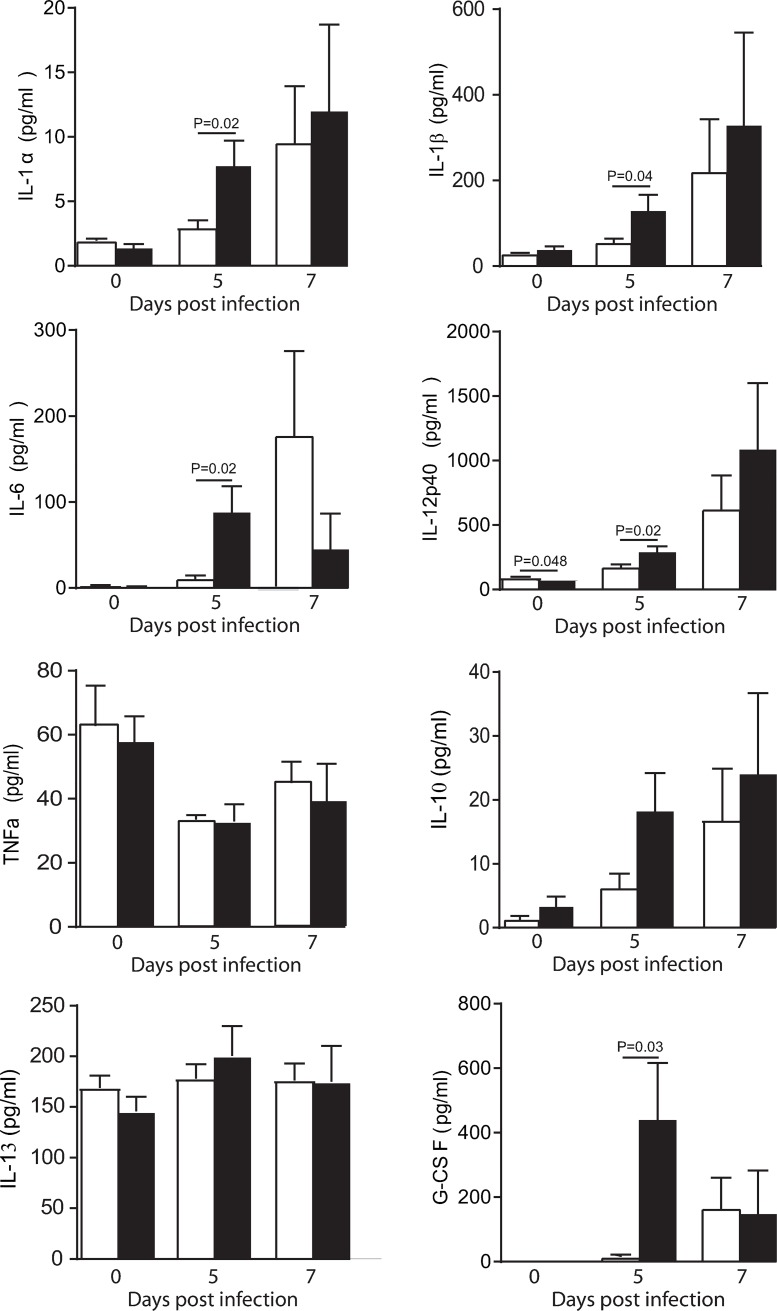
Impact of MBL deficiency on levels of cytokines in brain homogenates of mice infected with HSV-1. Levels of cytokines were measured by magnetic bead-based immunoassay in brain homogenates of WT (white) and MBL-null (black) mice. Results represent the mean ± standard error of the mean of 3–5 mice per group and per time point. Statistical analyses were performed using an unpaired Student t-test.

**Fig 6:**
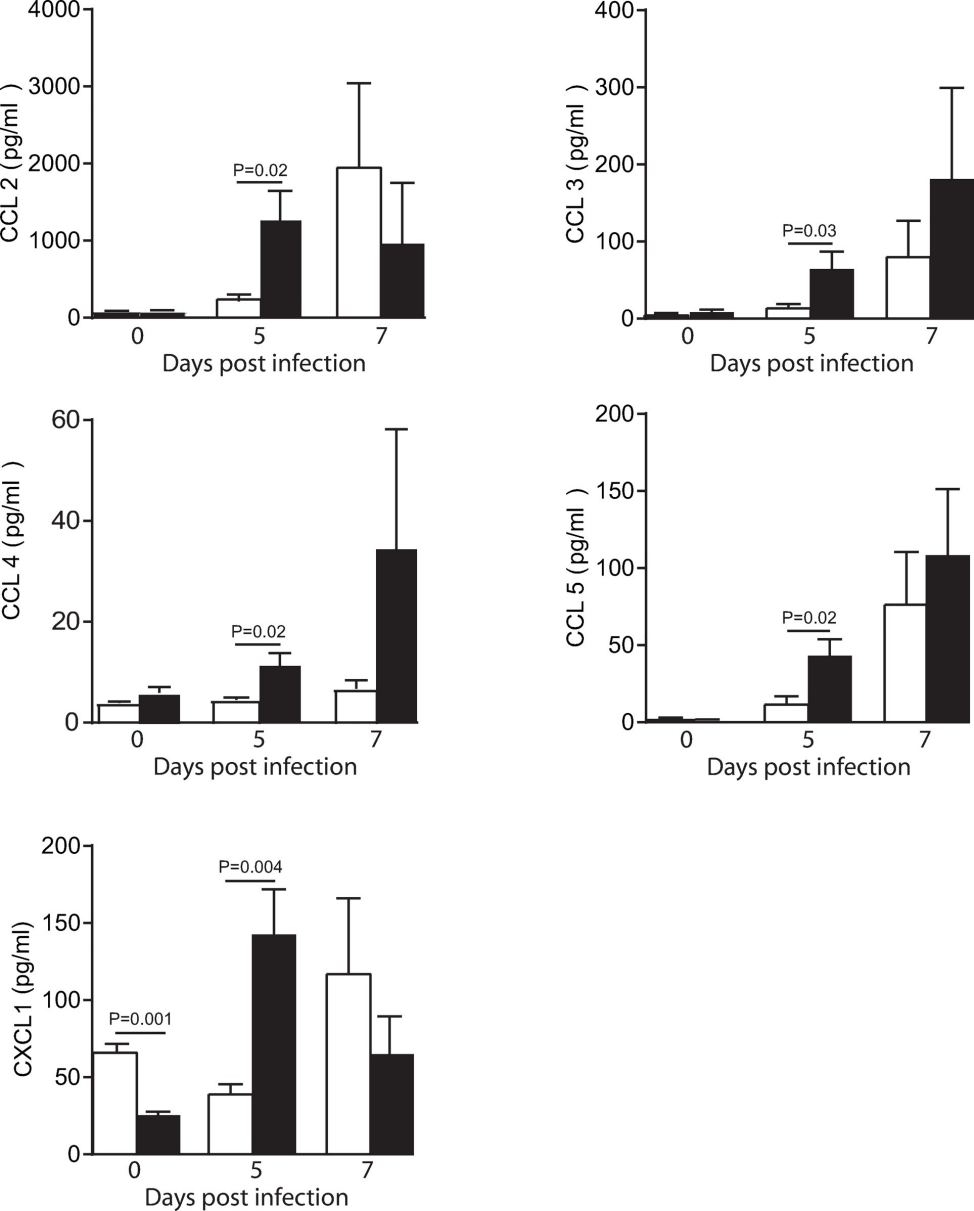
Impact of MBL deficiency on levels of chemokines in brain homogenates of mice infected with HSV-1. Levels of chemokines were measured by magnetic bead-based immunoassay in brain homogenates of WT (white) and MBL-null (black) mice. Results represent the mean ± standard error of the mean of 3–5 mice per group and per time point. Statistical analyses were performed using an unpaired Student t-test.

The inflammatory response among WT and MBL-null mice was compared by measuring pro-inflammatory (IL-1α, IL-1β, IL-6, IL-12p40 and TNF-α) and anti-inflammatory cytokines (IL-10, IL-13 and G-CSF, **Fig 5**) and chemokines (CCL2, CCL3, CCL4, CCL5 and CXCL1, **Fig 6**) levels in brain homogenates at different time points after HSV-1 infection. The levels of several pro-inflammatory cytokines (IL-1α, IL-1β, IL-6 and IL-12p40) and chemokines (CCL2, CCL3, CCL4 and CCL5), including agents with a neurotrophic or neuroprotective potential (G-CSF and CXCL1) were significantly increased in MBL-null mice compared to WT on day 5 post-infection (all P<0.05). There was no statistical differences between brain cytokines and chemokines levels on day 7 post-infection. No changes were observed in the levels of the anti-inflammatory cytokines (IL-10 and IL-13) and the pro-inflammatory cytokines TNF-α in brain homogenates of WT and MBL-null mice after infection with HSV-1 (**Fig 5**). Altogether, the increased brain viral load observed on day 5 post-infection in MBL-null mice may lead to an exaggerated inflammatory response.

## Discussion

In this study, we report two cases of adult HSE occurring in patients carrying very rare variants in MASP-2, a key protease in the lectin pathway of the complement system. A number of studies have shown that the complement is an important component of immune defences against HSV-1 [23, 24]. Deficiencies in different proteins of the complement such as C3 and C4 have been associated with impaired viral neutralization and reduced antibody production in mouse models of HSV-1 infection [25, 26]. Furthermore, it was clearly demonstrated that HSV-1 has developed escape strategies to avoid its neutralization by complement proteins [27]. Escape from the classical pathway occurs through an HSV-1 encoded receptor for the Fc domain of IgG (FcγR) which protects the virus from antibody-dependent complement neutralization [28]. Escape from the alternative pathway occurs through the HSV-1 encoded glycoprotein gC which protects the virus from destruction by complement in vitro [29–32] as well as in guinea pig models [33].

Fewer studies analysed the role of the lectin pathway in HSV-1 detection. The lectin pathway activity is triggered by recognition of mannan and carbohydrate structures present on surface of invading microorganisms [33–35] by pattern recognition receptors such as mannose-binding lectin, ficolins (L-ficolin, M-ficolin, H-ficolin) or collectins (CL-10 and CL-11), in complexes with MBL-associated serine protease 2 [36–38]. Specifically, MBL-1 and/or MBL-2 are capable to recognize structures present on HSV-1, as demonstrated in experiments using serum from mice and rats [39]. MASP-2 represents the enzymatic constituent of the lectin pathway and plays a fundamental role in both C4 and C2 cleavage [40].

Variants in *MASP2* leading to functional deficiencies have been reported [41, 42] but their association with diseases so far have been subject of controversy [41, 43–47]. In humans, relatively common polymorphisms within genes involved in the lectin pathway were associated with infectious and autoimmune diseases [43, 48–51]. Due to the ability of MBL to bind to a broad variety of fungi, bacteria and viruses, the lectin pathway contributes to different infections, pathogenesis and disease severity in mice [19, 52] and humans [53, 54]. Complement proteins are constitutively synthesized by different cells of the CNS including cells of the blood brain barrier (BBB), neurons and glial cells [55–57]. Since the CNS is considered to be an immune-privileged site, separated from circulating plasma complement components by the BBB, local complement proteins may be particularly relevant to viral immunity in the brain. The lectin complement pathway may be critical for HSV-1 immune detection, as an immune mediator whose specific role becomes obvious when other potentially redundant effectors are missing, as it is the case in chemotherapy-related neutropenic patients [58] or in newborns [59, 60]. This may be particularly relevant during the early steps of infection when the permeability and the integrity of the BBB is not compromised, still preventing penetration of antibodies into the CNS compartment.

The mutations observed in our adult HSE patients were associated with an abnormal protein secretion, a lost ability of auto-activation (G634R) and a reduced antiviral activity (G634R). At the population level, the proportion of very rare variants in *MASP2* was enriched among HSE patients compared to controls. By using a relevant intranasal HSE model [61], we showed that mice deficient in MBL had lower survival rates, higher viral load and a faster increase in the levels of cytokines and chemokines in the brain compared to WT mice. Murine models of HSE that consist in a primary and acute infection may not accurately reflect the pathology in humans (which is mainly due to virus reactivation). However, these data suggest that a deficiency in the lectin pathway results in a lack of control of viral replication leading to an exacerbated inflammatory response in the brain, that results in an increased mortality rate in mice infected with HSV-1. The mouse model of intranasal infection used in this study is not expected to induce HSV-1 viremia and spread to internal organs in WT animals [62, 63]; yet, one cannot exclude that such spread would have been observed in MBL deficient mice. MASP-2 deficient mice were protected by a patent and not available for this study, so that MBL-A x MBL-C deficient mice (only one MBL gene is expressed in humans whereas two closely related proteins MBL-A and MBL-C are produced in mice) were used instead of MASP-2 deficient mice to test the role of the lectin pathway in susceptibility to HSE. Since MBL is one of several pattern-recognition molecules associated with MASP-2, we believe that our experiment may have at worst underestimated the role of this pathway in susceptibility to HSE. Thus, the increased mortality observed in MBL-null mice compared to WT animals further confirms the protective role of the lectin pathway against HSE.

Previous studies mainly performed in children have shown that HSE is linked with mutations in genes involved in the TLR3/IFN immune pathway [10–17, 64, 65]. The role of this pathway is further supported in intranasal models of HSE using mice deficient in TRIF (the TLR3 adaptor [66]), Unc93B (protein involved in TLR3, TLR7 and TLR9 sorting [67]) and the IFN-α receptor [67]. Since HSE patients were relatively resistant to infectious diseases outside the CNS [68, 69], the TLR3/IFN pathway was proposed to be essential and non-redundant for CNS immunity against HSV-1 [70]. Yet, increasing evidences suggest that variants in immune pathways other than the TLR3-IFN axis may contribute to HSE [63, 71]. Functional impairment in the TLR3-IFN pathway could explain susceptibility to HSE in only 5% of affected children [15], in particular those with HSE recurrence, suggesting that other immune pathways may be involved in susceptibility to HSE in children. This may be even more relevant for adult patients, as HSE mainly results from reactivation of a latent virus in trigeminal ganglia neurons [3] contrasting with children where disease usually results from primary infection. Indeed, mutations in Tyrosine Kinase 2 (TYK2, [17]), Mitochondrial antiviral signaling protein (MAVS, [17]) have recently been described in adult HSE patients.

Altogether, this study shows that rare variants in MASP-2 may increase susceptibility to HSE, thereby suggesting that HSE does not solely result from mutations in genes of TLR3-IFN pathway. Further studies showing additional HSE-associated variants in the lectin pathway would reinforce these observations, as the disease in adults may result from a combination of conditions including genetic or acquired immune deficiency, timing of infection/reactivation and environmental factors.

## Material and methods

### Ethics statement

Patients and healthy volunteers included in this study signed an informed consent form for genetic and functional testing. The study was approved by the Cantonal Ethics Committee of the state of Vaud (CER-VD #130/08). All animals were used in accordance to the Canadian Council on Animal Care guidelines and the protocol was approved by the Animal Care Ethics Committee of Laval University (protocol no. 2013078).

### Study patients

A total of 15 pure European ancestry patients with a positive PCR for HSV-1 and signs/symptoms of HSE were included in the study. Samples from an additional patient with multiple sclerosis and family members (see results section) were included in functional experiments, allowing for G634R characterization in a total of 4 heterozygous carriers.

### MBL genotyping

Polymorphisms in the MBL promoter region (rs11003125 or H.L variant, rs7096206 or X, Y variant and rs7095891 or P, Q variant) were determined by sequencing PCR-amplified gDNA loci. Variants located in the first exon of the gene (rs5030737 or R52C or variant allele D, rs1800450 or G54D or variant allele B, rs1800451 or G57E or variant allele C) were determined by exome sequencing. The D, B and C variant alleles were collectively called O and the major alleles at these loci named A. The O indicated the presence of one or more mutant allele(s) in any of the 3 polymorphisms.

### Whole exome sequencing

Genomic DNA was extracted from venous blood samples of 15 cases of adult with HSE and 1 case of adult MS, all of pure European ancestry. Construction of paired-end sequence libraries and exome capture were performed by using 50Mb Agilent SureSelect Capture Kit. Captured libraries were sequenced on an Illumina platform as paired-end 54-bp reads according to the manufacturer's protocol. The purity filtered reads from the standard Illumina processing pipeline were aligned to the NCBI37 human reference genome using Novoalign (V3.08.00, Novocraft Technologies Sdn Bhd, Selangor, Malaysia), generating a binary alignment map (BAM) file per sample for each aligner. Single nucleotide variants (SNVs) and indels were called using the genome analyzer toolkit (GATK) v3.8 [72] and filtered using a minimum read depth (>10), minimum genotype quality (GQ>50), minimum alternative allele ratio (>0.3), minimum quality by depth (QD>5) and maximum FisherStrand (FS<60).

### Gene-burden analysis

Individuals of non-Finnish European (NFE) ancestry (15 cases and 294 in-house controls) were identified by using the Ethseq software [73]. Rare and deleterious variants were selected according to the following criteria: (i) allele frequency <0.001, as reported in the ExAC-NFE and gnomAD-NFE, (ii) nonsynonymous effect on the predicted protein product (missense, nonsense, frameshift, or essential splicing), and (iii) combined annotation dependant (CADD) score higher than 20 (representing the top 1% most deleterious variants according to the predictor, https://cadd.gs.washington.edu/info). A gene burden test was then performed in cases vs controls, by focusing on genes involved in innate immune response (gene ontology term GO:0045087) and by using the Fisher’s exact test to compute every associated p-value. Enrichment versus gnomAD-NFE population was done using variants selected by the same criteria as described above and Fisher's exact test was performed on an estimated gnomAD-NFE population of 60.000 individuals. Common MBL2 variants NM_000242.2:c.C154T:p.R52C, NM_000242.2:c.G161A:p.G54D and NM_000242.2:c.G170A:p.G57E were analysed. Analysis on genes related to the TLR3/IFN pathway concentrated on *UNC93B1*, *TLR3*, *TICAM1*, *TBK1*, *TRAF3*, *STAT1* and *IRF3*.

### Molecular dynamics simulations

MD simulations were performed with GROMACS [74, 75] version 2018.3 in periodic boundary conditions, using the all-atom CHARMM27 force field [76] and the TIP3P water model. The number of Na^+^ and Cl^-^ ions in solution was adjusted to neutralize the system and reach the physiological concentrations of 0.154 M. Before starting the MD simulations, missing residues and loops were modelled using the Dunbrack rotamer libraries [77] and the Modeller program [78]. Titratable side chains were protonated so as to allow hydrogen bonds with neighbouring residues. Electrostatic interactions were calculated with the Ewald particle-mesh method with a grid spacing of 1.2 Å. A cut-off of 12 Å was applied for the real-space Coulomb and van der Waals interactions. Bonds involving hydrogen atoms were constrained using the P-LINCS algorithm [75].The system was coupled to a Parinello-Rahman barostat with a relaxation time of 1 ps. The solute and the solvent were separately coupled to two nose-hoover thermostats, each with a relaxation time of 0.2 ps. A time integration step of 2 fs was used, with a temperature of 300 K and a pressure of 1 bar during the production trajectory. Initial structures were taken from the experimental 3D structure of the zymogen catalytic region of human MASP-2 (PDB ID 1ZJK [79]). Mutation G634R was introduced using the *swapaa* command of UCSF Chimera [80] v 1.12 and the Dunbrack backbone-dependent rotamer library [77]. Initial structures were energy optimized, heated from 0 to 300 K in 0.4 ns, equilibrated for a further 1 ns restraining each solute non-hydrogen atom to its original position, and finally equilibrated for 2 ns without restraints before data collection. Three and 6 MD simulations were carried out for the WT and mutated MASP-2, respectively, to assess the reproducibility of the results. Each MD simulation had a production time of 140 ns, saving coordinates every 0.05 ns.

### Production of recombinant proteins

The human MBL as well as the WT and mutant MASP-2 cDNAs were amplified by PCR from total RNA isolated from whole blood. Purified products were subcloned into a pGEM-T Easy vector by T4 DNA ligase (Promega) and sequenced. We generated pcDNA3.1 expression vectors (Thermofisher) encoding the different MASP-2 variants and MBL. Plasmids containing cDNAs were used for the transient transfection of Hela cells. Briefly, plasmids were mixed with JetPei (Polyplus transfection, USA), according to the manufacturer’s instructions. Cells were cultured for 24 h, 48 h or 72 h in a medium deprived in Fetal Calf Serum (FCS). Supernatants were collected by centrifugation at 10,000 xg, 5 min, 4°C and cells were lysed with a lysis buffer containing Tris 10 mM, pH 8, NaCl 150 mM, NP-40 0.5%, NaF 10 mM, Na-orthovanadate 1 mM and protease inhibitors and collected by centrifugation at 10,000 xg, 5 min, 4°C.

### Expression of recombinant proteins

Cells were washed 2 times with phosphate-buffered saline (PBS), harvested with the lysis buffer previously described and centrifuged at 10,000 xg at 4°C, 5 min. For some experiments, cell culture supernatants were concentrated on Microcon concentration units (Amicon, Merck). Protein content was determined by the Biorad Protein assay (Biorad). Proteins (20 μg-40 μg) were then subjected to SDS-PAGE under reducing and non- reducing conditions and then transferred onto a nitrocellulose membrane. The rabbit anti-human MASP-2 H-60 antibody (1/200, sc-50420, Santa Cruz Biotechnology), rabbit anti-human MBL-2 antibody (1/1,000, orb 31822 Biorbyt), rabbit anti-human macrophage migration inhibitory factor (MIF) antibody [81] and mouse anti-B actin antibody (1/1,000, sc-8432, Santa Cruz Biotechnology) were used as primary antibodies and were detected using anti-rabbit or mouse IgG antibodies conjugated to horseradish peroxidase (1/10,000, Amersham Biosciences). Detection was done with the ECL chemiluminescence kit (Pierce) according to the manufacturer’s protocol.

### MASP-2 dosage

MASP-2 concentrations were measured in the plasma, cell lysates or cell culture supernatants using enzyme-linked immunosorbent assay (HK326, Hycult biotechnology, Uden, The Netherlands) according to the manufacturer’s instructions.

### MBL/MASP-2 dosage

The *in vitro* quantitative determination of functional human MBL/MASP-2 in the plasma was measured by enzyme-linked immunosorbent assay (HK327, Hycult biotechnology, Uden, The Netherlands) according to the manufacturer’s instructions. This assay quantifies the ability of MBL/MASP-2 complexes to initiate C4 cleavage when bound to mannan.

### Immunoprecipitation

Supernatants from WT- or mutant MASP-2-expressing cells were mixed with an equal volume of supernatants from MBL-expressing cells. After 5 h incubation in a buffer containing Tris 25mM, pH 8, NaCl 150mM, CaCl2 5mM, at 4°C, immunoprecipitation of MBL under non-denaturing conditions was performed with 3 μl of mouse anti-human MBL2 antibody (ab26277, abcam). Proteins were then resolved by SDS-PAGE and then transferred onto a nitrocellulose membrane. The association of MASP-2 was revealed by using a rabbit anti-human MASP-2 H-60 antibody (1/200, sc-50420, Santa Cruz Biotechnology). The expression of MBL was also determined with a rabbit anti-human MBL-2 antibody (1/1,000, orb 31822 Biorbyt).

### HSV-1 amplification

Human herpesvirus 1 strain MacIntyre used in viral neutralization studies was purchased from ATCC (ATCC VR-539) and was amplified on a 80% confluency African green monkey kidney (Vero) cell line in DMEM/GlutaMax supplemented with 10% FBS. Briefly, Vero cells were infected with HSV-1 at a multiplicity of infection of 0.01, during at least 2h at 37°C. Medium was then replaced and supernatant was collected after 4 days incubation at 37°C and clarified at 4,500 xg for 30 min. HSV-1 titers were determined by plaque assay on Vero cells and virus preparation was aliquoted and stored at -80°C.

### Plaque assay

Different dilutions of HSV-1 suspension were incubated on Vero cells during at least 2h at 37°C. Supernatant was then aspirated and cells washed with PBS. Cells were then incubated in DMEM containing 1% MethylCellulose and 0.33% Sodium Bicarbonate at 37°C for several days, until the plaque formation. Medium was then aspirated and the cells were fixed and stained with a mixture of Paraformaldehyde 2% and crystal violet 0.5% for 15 min at room temperature. HSV-1 titre was evaluated from plaque counting and expressed as PFU/ml.

### Antibodies deprived plasma

Plasma samples were 2-fold diluted in Sodium phosphate 20mM, pH 7 and added to protein G HP SpinTrap columns (GE Healthcare life Sciences, United Kingdom) according to the manufacturer’s instructions. Antibodies were attached to the Protein G Sepharose and flow-through containing antibodies deprived plasma was collected and immediately stored at 4°C.

### HSV-1 neutralization

Antibodies deprived plasma were mixed with different dilutions of HSV-1 during 20 min at 37°C. The mixture was then incubated on Vero cells during at least 2h at 37°C and the neutralizing activity of plasma was determined using a plaque assay on Vero cells.

### Animals

Mice homozygous for MBL-AxMBL-C knockout mutations (MBL-null; B6.129S4-*Mbl1*^*tm1Kata*^ *Mbl2*^*tm1Kata*^/J) and wild-type C57BL/6J mice were purchased from Jackson Laboratory (Bar Harbour). Animals were housed three to five per cage and acclimated to standard laboratory conditions for one week.

In two sets of experiments, 10 MBL-null and 10 WT C57BL/6J male mice at 6–7 weeks of age were infected intranasally with 700,000 PFUs of the neurovirulent clinical HSV-1 strain H25 in 20 μl of minimal essential medium (MEM) [61]. Mice were monitored for weight loss, neurological signs (shaking movement, hind limb paralysis, prostration and convulsion), ruffled fur, ocular swelling and survival rate over 18 days. Animals were sacrificed when a weight loss equal to or greater than 20% or a combination of two other obvious sickness signs were recorded. The results of the two experiments were pooled for analysis.

In a third set of experiments, MBL-null and WT C57BL/6J male mice at 6–7 weeks of age were infected intranasally with 700,000 PFUs of HSV-1 strain H25 in 20 μl of MEM. Mice were sacrificed prior to infection (day 0; n = 3 for both mouse strains) and on days 5 (n = 5 for both mouse strains) and 7 (n = 4 for both mouse strains) post-infection. Mice were euthanized and sacrificed by intracardiac perfusion with cold 0.9% saline. Brain was harvested and homogenized in 700 μl PBS containing protease and phosphatase inhibitor cocktails (Roche diagnostics) with a homogenizer (BioSpec Products, Inc.). For quantitative RT-PCR determinations of IFN-α/–β mRNA levels, 100 μl of brain homogenate were added to 1 ml of TRIzol reagent (Invitrogen). All samples were maintained on dry ice and stored at -80°C until used.

Total DNA was extracted from 10 mg of brain homogenates with the MagNA Pure LC DNA Isolation Kit II (Tissue; Roche Molecular Systems) according to the instructions of the manufacturer and eluted in 200 μl of elution buffer. Real-time qPCR assay was performed in duplicate using 5 μl of extracted total DNA mixed with LightCycler 480 Probes Master on a LightCycler 480 system (both from Roche Molecular System) and external standards were run in parallel as previously described [82]. Primers and probes targeted a conserved region of the DNA polymerase of HSV-1. The limit of detection of the assay is 200 copies of viral genome/mg of brain.

Brain homogenates were centrifuged at 10,000 *xg* for 10 min at 4°C. A volume of 50 μl of supernatant was taken for the determination of cytokine and chemokine levels using a commercial multiplex mouse cytokine magnetic bead-based immunoassay (Bio-Plex Pro Mouse Cytokine 23-plex Assay; Bio-Rad Laboratories) according to the manufacturer’s instructions. Mean fluorescence intensity from all the bead combinations tested was analyzed by the Bio-Plex Manager Software v6.0 (Bio-Rad Laboratories).

For the determination of IFN-α/–β transcripts by RT-qPCR, total RNA was extracted from brain homogenates using Direct-zol RNA MiniPrep Plus Kit (Zymo Research Corporation) as described in the manufacturer’s instructions. Complementary DNAs were generated using a random primer hexamer (Invitrogen) and the SuperScript II polymerase (Invitrogen). Amplicons were detected using the Amplifluor Uniprimer system in which forward primers contained the 5’ Z sequence ACT-G-A-A-C-C-T-G-A-C-C-G-T-ACA. Amplification efficiencies were validated and normalized to that of the 18S ribosomal subunit gene. The different forward and reverse primers used are described in S1 Table. Real-time qPCR was performed in 50 μl volume containing 25 μl of 2x Universal PCR Master Mix (Applied Biosystems), 10 nmol/l of Z-tail forward primer, 100 nmol/l of untail reverse primer (both from Integrated DNA Technologies), 100 nmol/l of Amplifluor Uniprimer probe (Sigma-Aldrich). The mixture was incubated at 50°C for 2 min, at 95°C for 4 min and then cycled 55-times at 95°C for 15 s and at 55°C for 40 s using the Applied Biosystems prism 7900 Sequence Detector (Applied Biosystems). An external standard was run in parallel.

Differences in mouse survival rates were compared using a log-rank (Mantel-Cox) test. Differences in viral load, cytokine and chemokine production and IFN-α/–β mRNA levels were evaluated using an unpaired Student t-test. All statistical analyses were performed using GraphPad Prism software program v5 (GraphPad Software, San Diego, CA). A *P* value ≤ 0.05 was considered as statistically significant.

## Supporting information

S1 Fig Running average of the minimal distance between His483 and Asp532, during the production part of the 3 MD simulations of the wild-type system (in blue) and the 6 MD simulations of the G634R mutant (in brown). (TIF)

S2 Fig Running average of the minimal distance between His483 and Ser633, during the production part of the 3 MD simulations of the wild-type system (in blue) and of the 6 MD simulations of the G634R mutant (in brown). (TIF)

S3 Fig Sequence alignment of MASP-2 from 19 organisms, in the region of Arg203. The residue numbering provided above the sequences corresponds to the human protein. Arg203 is in the middle of the very conserved 199-PEYPxPYPK-207 motif. The sequence alignement was performed with the MUSCLE program.
(TIF)

S4 Fig Experimental structure of the EGF-like and CUB2 domains of MASP-1 (PDB ID 4AQB) [PMID: 22854970]. Asn204 of MASP-1 (corresponding to Arg203 of MASP-2) is shown in ball and stick representation. All other residues of the 200-PDFPxPYPK-208 motif of MASP-1 are shown as thick lines. This motif is creating a non-polar cluster that stabilizes the structure of MASP-1 in this region. The corresponding MASP-2 motif 199-PEYPxPYPK-207 is expected to play the same role in MASP-2. Residue Asn204 in MASP-1 (respectively Arg203 in MASP-2) is facing the solvent and possibly exchanges a hydrogen bond with Asp201 (respectively Glu202 in MASP-2). Replacing this residue by a non-polar and aromatic tryptophan residue is expected to impact the structural stability of MASP-1 (respectively MASP-2).
(TIF)

S5 Fig Survival rates of WT and MBL-null (dotted line) mice. Results represent data from two separate experiments with 10 WT and 10 MBL-null mice each.
(TIF)

S1 Table Sequence of forward and reverse primers used to determine the levels of IFN-α/- β mRNAs by RT-qPCR. (PDF)
