# Supplementary figures and images for: Herpes simplex encephalitis in adult patients with MASP-2 deficiency
Source: PLoS Pathog. 2019 Dec 23;15(12):e1008168. doi: 10.1371/journal.ppat.1008168 (PMC6944389; doi:10.1371/journal.ppat.1008168)

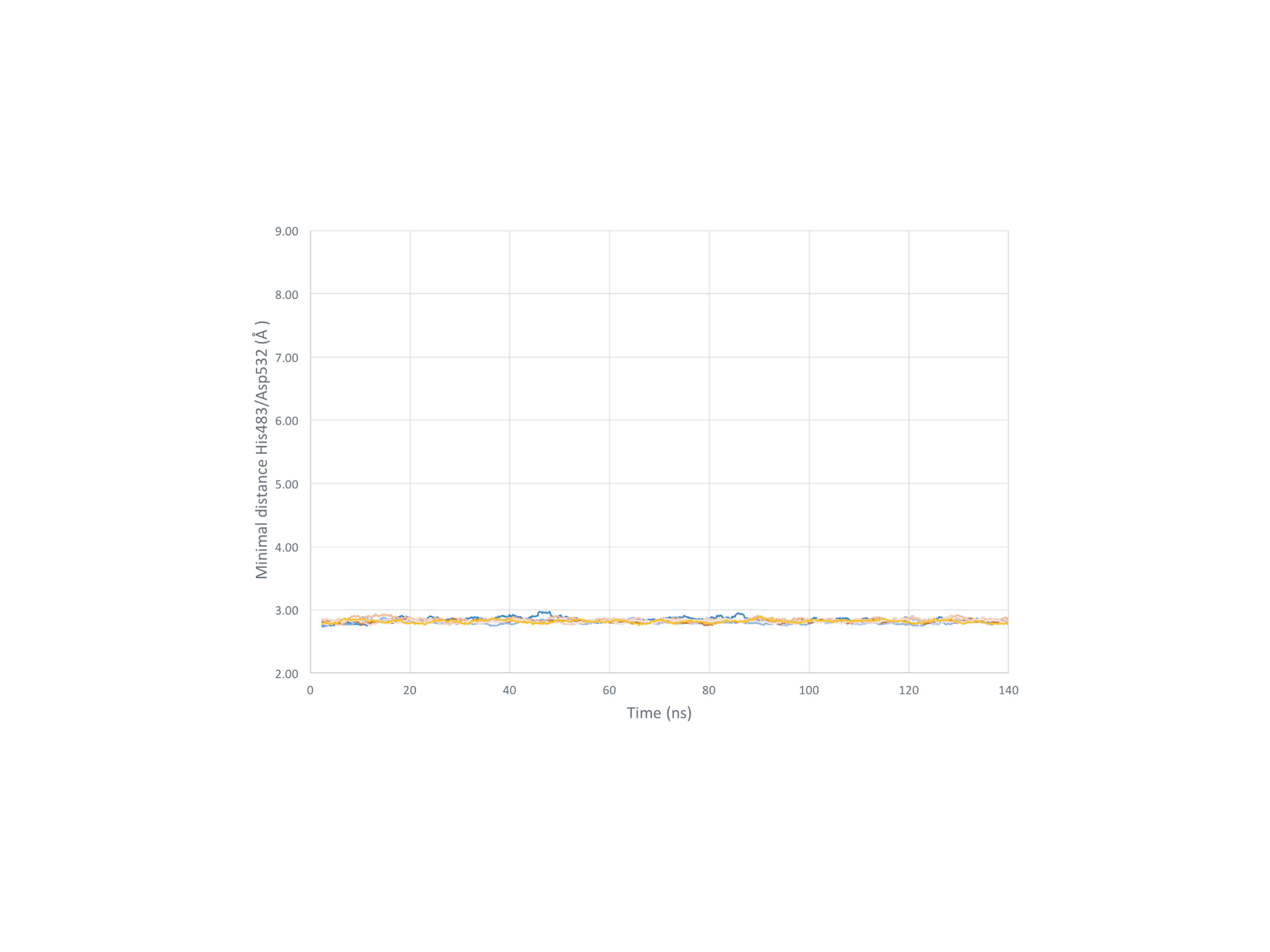

Supplement: S1 Fig — (TIF) [file ppat.1008168.s001.tif]

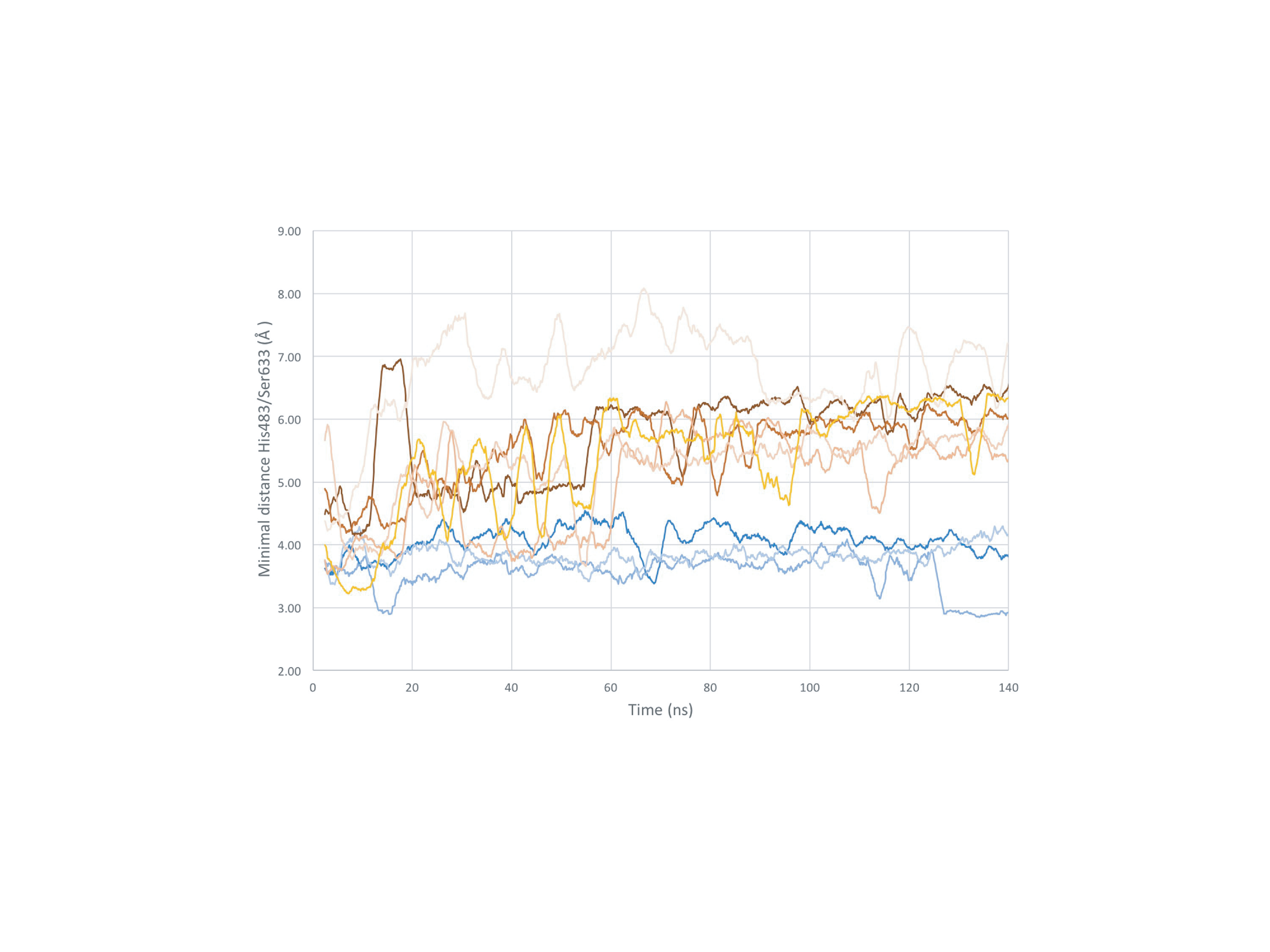

Supplement: S2 Fig — (TIF) [file ppat.1008168.s002.tif]

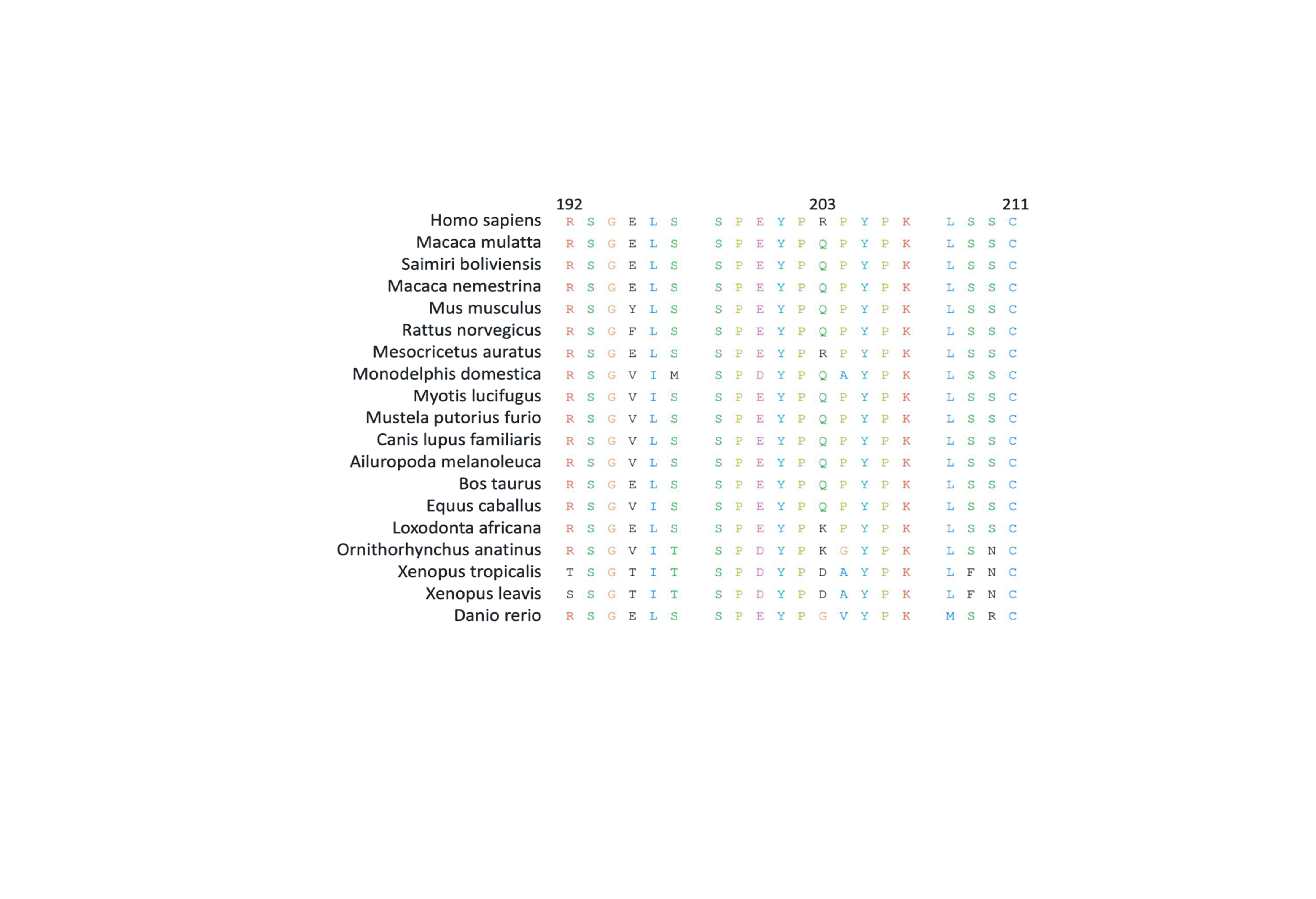

Supplement: S3 Fig — The residue numbering provided above the sequences corresponds to the human protein. Arg203 is in the middle of the very conserved 199-PEYPxPYPK-207 motif. The sequence alignement was performed with the MUSCLE program. (TIF) [file ppat.1008168.s003.tif]

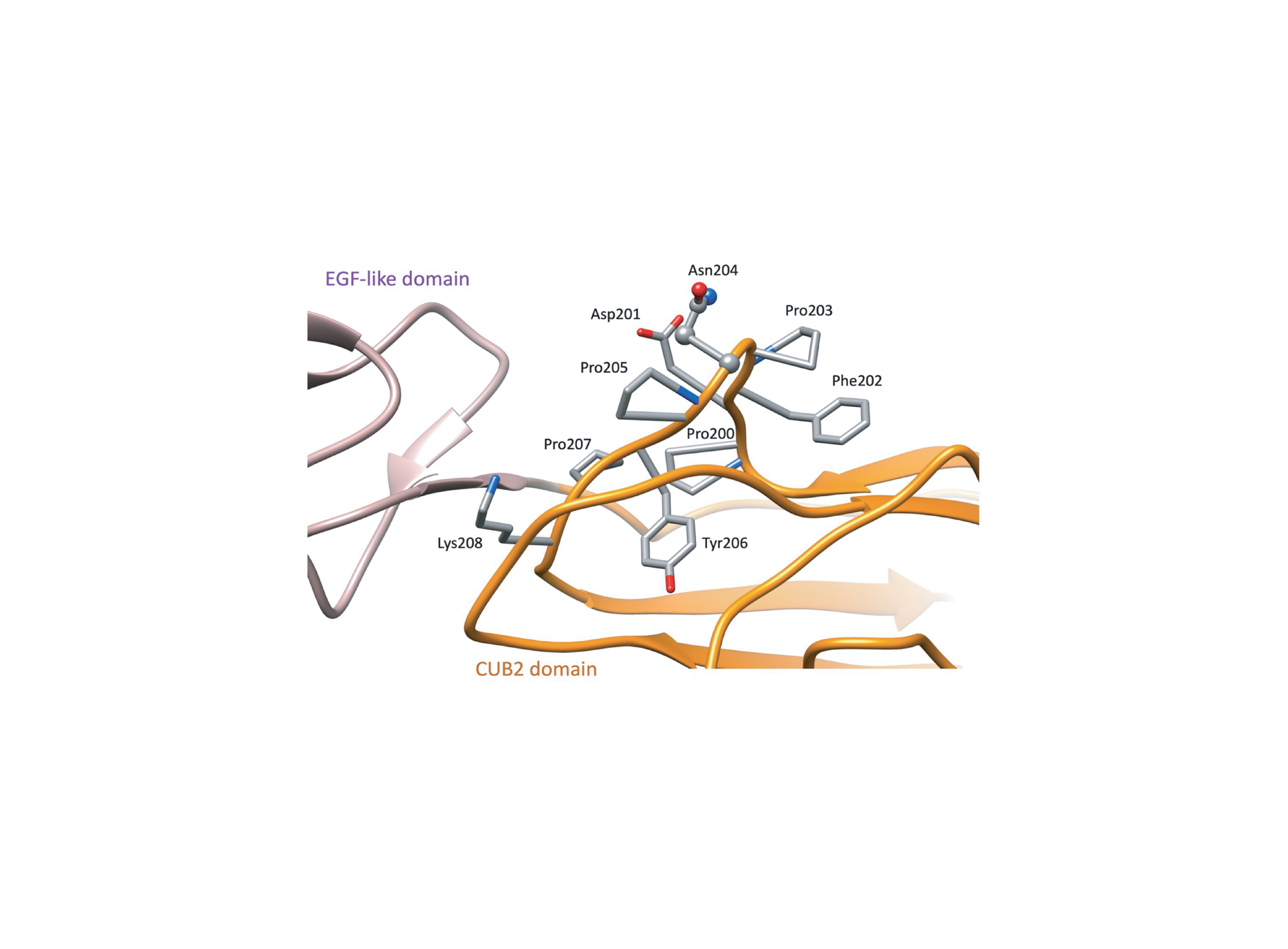

Supplement: S4 Fig — Asn204 of MASP-1 (corresponding to Arg203 of MASP-2) is shown in ball and stick representation. All other residues of the 200-PDFPxPYPK-208 motif of MASP-1 are shown as thick lines. This motif is creating a non-polar cluster that stabilizes the structure of MASP-1 in this region. The corresponding MASP-2 motif 199-PEYPxPYPK-207 is expected to play the same role in MASP-2. Residue Asn204 in MASP-1 (respectively Arg203 in MASP-2) is facing the solvent and possibly exchanges a hydrogen bond with Asp201 (respectively Glu202 in MASP-2). Replacing this residue by a non-polar and aromatic tryptophan residue is expected to impact the structural stability of MASP-1 (respectively MASP-2). (TIF) [file ppat.1008168.s004.tif]

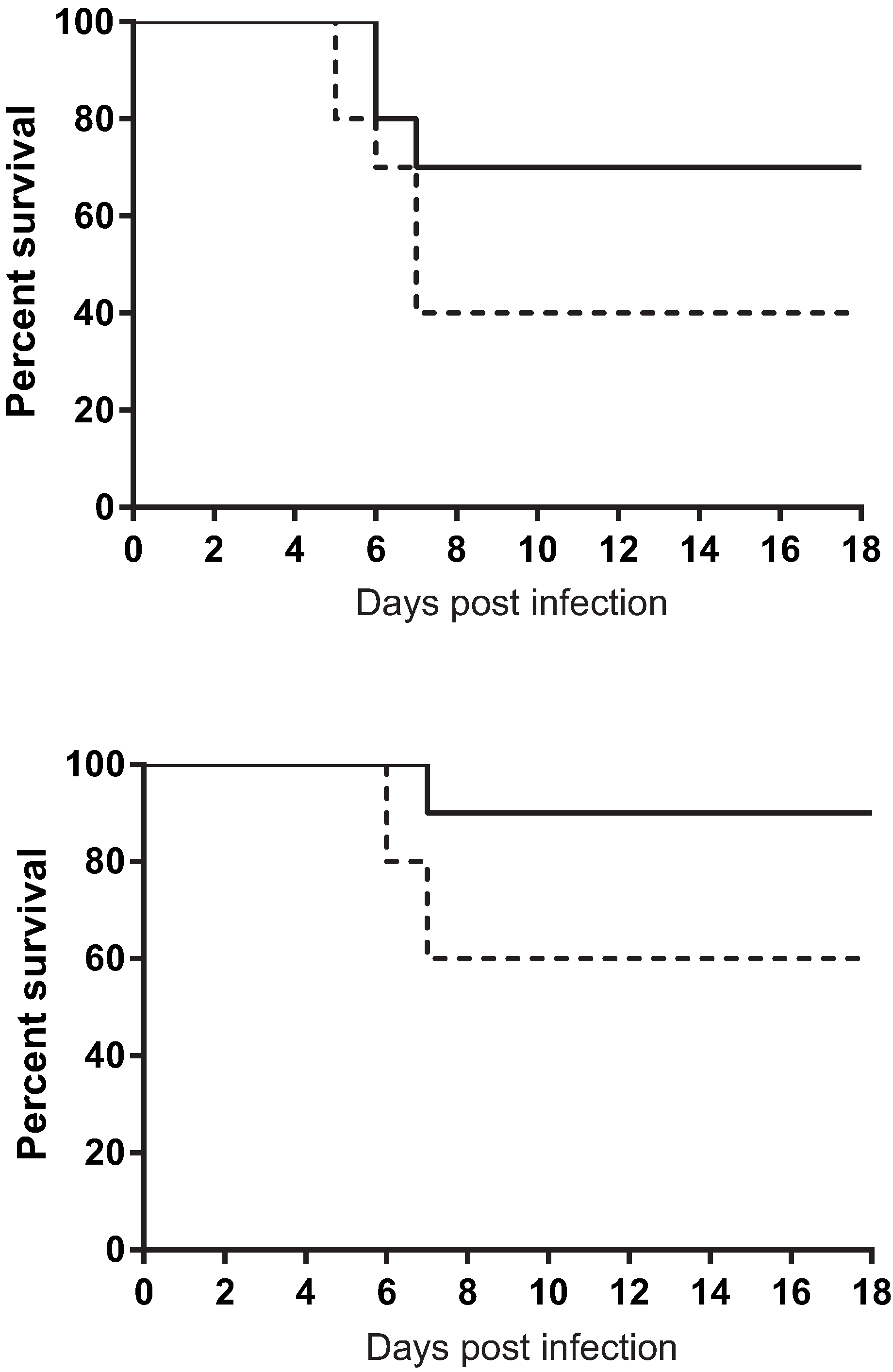

Supplement: S5 Fig — Results represent data from two separate experiments with 10 WT and 10 MBL-null mice each. (TIF) [file ppat.1008168.s005.tif]
