# Supplementary material for: Herpes simplex encephalitis in adult patients with MASP-2 deficiency
Source: PLoS Pathog. 2019 Dec 23;15(12):e1008168. doi: 10.1371/journal.ppat.1008168 (PMC6944389; doi:10.1371/journal.ppat.1008168)
Supplement: S1 Table — (PDF) [file ppat.1008168.s006.pdf]

Primer	Sequence
18S ribosomal subunit Reverse	AATGAGCCATTCGCAGTTTCA
18S ribosomal subunit Forward	TGCATGTCTAAGTACGCACGG
IFN-α Reverse	TTCTGCTCTGACCACCTCCC
IFN-α Forward	TTTCCACAGGATCACTGTGTACCT
IFN-β Reverse	GGCGTAGCTGTTGTACTTCATGA
IFN-β Forward	TACTGGAGGGTGCAAAGGTACC
